# Efficient Generation of Large Collections of Metal–Organic Framework Structures Containing Well-Defined Point Defects

**DOI:** 10.1021/acs.jpclett.3c01524

**Published:** 2023-07-18

**Authors:** Zhenzi Yu, Shubham Jamdade, Xiaohan Yu, Xuqing Cai, David S. Sholl

**Affiliations:** † School of Chemical & Biomolecular Engineering, Georgia Institute of Technology, Atlanta, Georgia 30332, United States; ‡ Oak Ridge National Laboratory, Oak Ridge, Tennessee 37830, United States

## Abstract

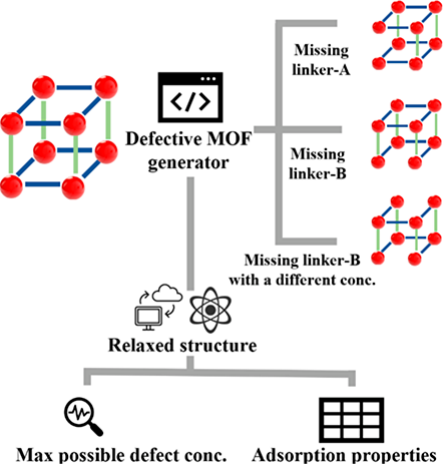

High-throughput molecular simulations of metal–organic frameworks (MOFs) are a useful complement to experiments to identify candidates for chemical separation and storage. All previous efforts of this kind have used simulations in which MOFs are approximated as defect-free. We introduce a tool to readily generate missing-linker defects in MOFs and demonstrate this tool with a collection of 507 defective MOFs. We introduce the concept of the maximum possible defect concentration; at higher defect concentrations, deviations from the defect-free crystal structure would be readily evident experimentally. We studied the impact of defects on molecular adsorption as a function of defect concentrations. Defects have a slightly negative or negligible influence on adsorption at low pressures for ethene, ethane, and CO_2_ but a strong positive influence for methanol due to hydrogen bonding with defects. Defective structures tend to have loadings slightly higher than those of defect-free structures for all adsorbates at elevated pressures.

Metal–organic frameworks (MOFs) are made up of metal clusters and organic linkers that have useful characteristics such as high porosity, large surface areas, and varied chemical environments within their pores.^1−4^ Tens of thousands of MOF structures have been reported experimentally, and large collections of crystal structures have been used for high-throughput screening studies. For example, molecular simulation techniques have been used to predict how diverse collections of molecules adsorb in large numbers of MOFs, and machine learning methods have been applied to expand these predictions.^5−7^ All high-throughput computational studies of adsorption in MOFs to date, however, rely on the assumption that the MOF crystal structures of interest are defect-free.

As in all materials, defects in MOFs are ubiquitous and unavoidable.^8^ Defects can occur for various reasons during synthesis or during the activation of materials to remove solvents from a MOF’s pores. In addition, defects can also be intentionally introduced into MOF structures in a controlled manner via so-called defect engineering.^9−12^ MOF defects can exist as spatially extended defects or as point defects. It is typically challenging to characterize defects in MOFs experimentally, especially in “clean” materials where the density of defects is expected to be low. There are, however, some examples in which characterization tools have been used to probe point defects in MOFs. For example, Ren et al. used in situ IR and ex-situ solid-state NMR to understand the formation of point defects in different solvent systems.^13^ In another example, Yu et al. used UV–vis spectrometry, thermogravimetric analysis, and inductively coupled plasma mass spectrometry to study point defect sites in a MOF.^14^ Extended defects in MOFs are more difficult to detect since organic linkers are often destroyed by traditional electron microscopy techniques.^15,16^

Detailed theoretical calculations have been useful sources of information about point defects in MOFs. For example, Cui et al. recently used DFT and a microkinetic model to study the acid gas-induced defect propagation in ZIFs, giving insights into the autocatalytic nature of ZIF degradation and the spatial distribution of defects.^17^ This work relied on several previous studies that performed DFT calculations of various defect configurations in ZIFs.^18,19^

Regardless of whether they are present intrinsically or deliberately introduced, defects can in some cases significantly impact the properties of MOFs, affecting their stability, selectivity, diffusion, and adsorption capacity. Hossain et al. studied the impact of defects on CO_2_ and water adsorption in UiO-66 and found that defect sites have a greater influence on low-pressure CO_2_ adsorption in MOFs than the coadsorption of water, thus altering the selectivity of the MOF.^20^ In a study of water adsorption of DMOF-1 by Chen et al., the presence of defects explained the water intrusion phenomenon observed experimentally.^21^ Cai and Sholl showed that point defects in Zn(tbip) are responsible for the unexpected molecular diffusion behavior in this MOF’s 1D channels^22^ and that missing water defects play a pivotal role in the interesting separation phenomena that are possible with the MOF UTSA-280.^23^

Although computational studies of defects in MOFs have been reported, to date, these rely on structures that are modified from known pristine crystal structures one at a time, which is a time-consuming and labor-intensive process. This situation has prevented any of the methods that have been developed for the high-throughput computational modeling of MOFs from being extended to situations where defects might be important. In this work, we introduce a set of tools to generate MOFs containing point defects in an automated way from crystal structures and demonstrate these tools with a data set of hundreds of defective MOFs. We benchmark the resulting structures and pipeline using widely studied MOFs and then examine a set of 20 MOFs to address two questions related to defects that have not been probed before. First, we ask what maximum possible defect concentrations in each material are consistent with the structural data that are typically reported during MOF synthesis. Second, we use molecular simulations to understand how strongly the presence of defects influences the adsorption properties of a variety of possible adsorbing molecules as a function of molecular loading.

In this work, we focus only on point defects in MOFs, a class that includes missing metal cluster defects, dangling linker defects, and missing linker defects. Among these, missing linker defects have been the most carefully described in previous work.^17,24,25^ For this reason, we consider only the task of efficiently generating structures with missing linker defects. At least four variables can be defined to describe the presence of missing linker defects in an MOF structure: missing linker types, defect concentration, capping agents, and short-range order (SRO). Removing linkers in MOFs typically creates open metal sites (OMS). Depending on the synthesis or operation conditions, different capping agents can be coordinated with the OMS. Common capping agents include OH^–^, H_2_O, formate, and halogens.^26^ Without losing generality, we used OH^–^ or H_2_O as the capping agent for the high-throughput generation of defective structures, choosing the species that leads to charge neutrality when the capping agent replaces a missing linker. The structures generated with these capping agents could be generalized to include other capping agents or to present OMS without difficulty if the specific application for a material meant that alternative capping agents are appropriate. In MOFs that include more than one linker species, the resulting missing linker type defines which specific linker is removed to create a point defect. The defect concentration is defined as the molar ratio of the missing linker; for example, one missing linker out of ten linkers in a structure defines a defect concentration of 0.1. The relative spatial location of point defects could impact a MOF’s properties.^17,27^ Measures of SRO such as the Warren-Cowley parameter are likely to be useful in characterizing these kinds of effects.^28,29^ Systematically exploring SRO effects typically requires large computational volumes; therefore, in this paper, we have opted to consider only configurations that can be readily defined with relatively small computational supercells and periodic boundary conditions. As described in the Supporting Information, however, we have developed tools allowing SRO in large computational volumes to be controlled. Because the structures generated with our approach have periodic boundary conditions, the missing linker defects have long-range crystalline order, even in cases where large computational volumes are used.

We first picked HKUST-1, UiO-66, IRMOF-1, and ZIF-8, which are the most widely studied MOFs,^30^ as representative examples to benchmark our MOF structure generator and associated simulations. The defective structure generation process is illustrated in Figure 1, where UiO-66 is used as an example (see Supporting Information for more detail). In this case, 10 C_8_H_4_O_4_^2–^ groups in the computational volume were identified as linkers. Two linkers were randomly selected and removed. Because each of the removed linkers has a −2 formal charge, our approach selects OH^–^ and H_2_O as the capping agents for each end of each linker that is removed. The positions of the capping agents are defined by the vector-sum algorithm mentioned in the Supporting Information in the software implementation section. Tan et al. reported experimental evidence that COO^–^ and H_2_O are the capping agent in some treatments of UiO-66.^31^ Our capping choice is chemically similar and has also been used in multiple previous studies.^20,27,32^

**Figure 1:**
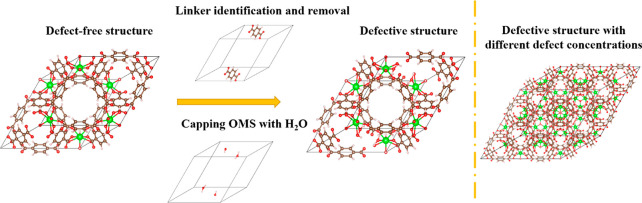
Illustration of the defective structure generation process for UiO-66.

Once it is straightforward to generate defective structures with varying concentrations of defects, it is interesting to ask what concentrations of defects are consistent with the structural information routinely available experimentally. To this end, we define a MOF’s maximum possible defect concentration (MPDC) as the largest defect concentration that does not cause significant changes in the crystallinity or surface area relative to the pristine material. We picked these two criteria because PXRD and BET surface area are standard experimental procedures that are widely reported to assess the synthesis of MOFs (see Supporting Information for more details). If multiple missing linker types are available, MPDC is defined by using the missing linker type that can achieve the maximum defect concentration.

Crystalline consistency was judged qualitatively by the absence of peak shifts and peak splitting in the simulated PXRD spectrum. To make this concept more quantitative, we defined PXRD similarity scores as shown in the Supporting Information. In our high-throughput pipeline, defective structures with PXRD similarity scores <0.8 and/or surface areas that differ from the pristine structure by more than 5% were considered to be readily distinguishable from the pristine structure. It is important to note that no universally accepted standards exist to determine the consistency of PXRD patterns and surface areas. Significant variations have been reported depending on the experimental setup or even data analysis.^33,34^ Thus, we recommend that users of our methods manually examine the PXRD patterns and surface area changes associated with the inclusion of defects to aid interpretation relative to the experimental data for specific materials of interest.

To illustrate the concept of the MPDC, we discuss this quantity in turn for each of the four representative MOFs listed above. For each defective structure, a full structural relaxation was performed using DFT (see Supporting Information for more details). For HKUST-1, numerous defect engineering studies have been conducted to tune sorption and catalytic properties.^9,35^ As shown in Figure 2a, starting from a defect concentration of 0.25, the PXRD peak at 2θ = 12° splits and multiple peaks shift relative to the pristine structure. Our calculations show that the MPDC for HKUST-1 is ∼0.12. Zhang et al. reported that HKUST-1 structures at different defect concentrations can be synthesized,^35^ but the exact concentration cannot be readily quantified during experiments. Our calculations provide some quantification of the possible defect concentrations in HKUST-1 for the first time.

**Figure 2:**
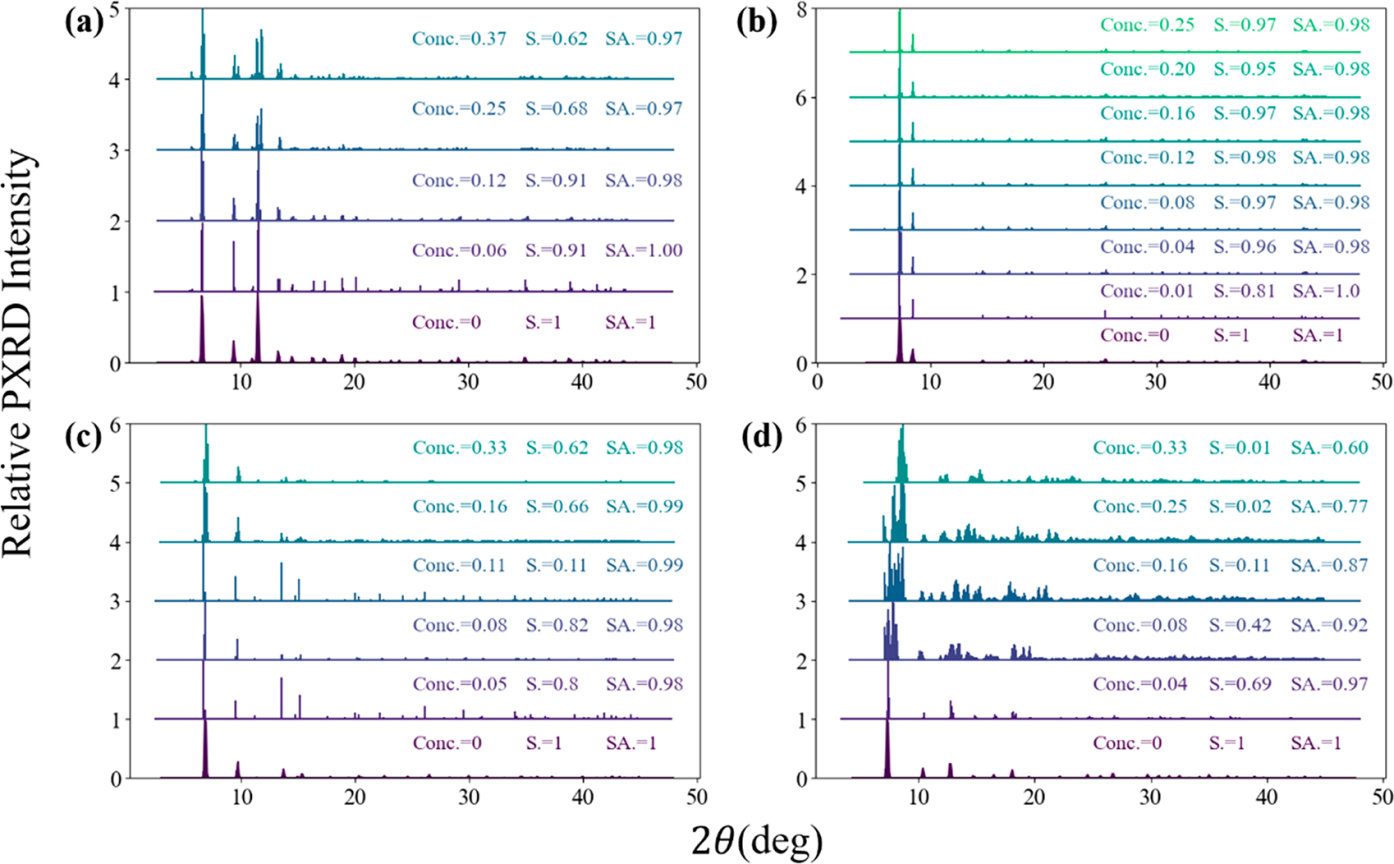
PXRD similarity score (S) and surface area ratio (SA) for four representative MOFs at different defect concentrations (Conc.): (a) HKUST-1, (b) UiO-66, (c) IRMOF-1, and (d) ZIF-8. All structures were DFT-optimized.

For UiO-66, a defect concentration of 0.1 has been widely reported experimentally in defect engineering studies, with concentrations up to 0.15 or even 0.4 also being reported with some unconventional synthesis routes.^24,36,37^ In these studies, no peak splits or shifts are observed for defective MOFs, but surface area changes of as high as 20% were reported. Our results in Figure 2b illustrate that having defect concentrations as high as 0.25 introduces minimal changes in the PXRD pattern and surface area, which is consistent with the experimental observation. The calculated surface areas for UiO-66 in Figure 2b do not change as defects are added because we picked helium as the default probe during the high-throughput process and used a geometry-based algorithm, which is not sensitive to the missing linkers when pores in MOFs are much larger than the probe. If N_2_ is used as the probe by repeating our surface area calculations using a probe size of 1.86 Å, then the surface area changes from 1093 m^2^/g for the defect-free structure to 1800 m^2^/g at a defect concentration of 0.25. Structures with higher defect concentrations for UiO-66 were not generated because a higher defect concentration caused the generation of covalently bonded fragments that are not connected by any bonds with the extended MOF structure. From our calculations, we conclude that the MPDC for UiO-66 is around 0.25.

For IRMOF-1, only conceptual modeling defect studies have been conducted, where a defect structure with a concentration of 0.27 was used without validation.^38^ Our results in Figure 2c, however, indicate that the MPDC for IRMOF-1 is 0.11. A combined experiment and simulation study indicated that periodic defects contribute to a triclinic distortion in ZIF-8 at a defect concentration of 1/24 or below.^39^ As shown in Figure 2d, even the smallest defect concentration we examined for ZIF-8, 0.04, introduces noticeable PXRD peak shifts and splits relative to the pristine structure. Structures with defect concentrations of 0.08 and higher show near zero PXRD similarity scores, indicating strong changes due to the defects. We conclude the MPDC for ZIF-8 is <0.04. Determining a more precise value for this MPDC was not possible because of the very large computational volumes that would have to be DFT optimized for lower defect concentrations.

As noted above, we generated defective structures for a total of 507 MOFs. By considering multiple defect concentrations for each MOF, we generated a total of 8323 structures. To probe the impact of defects on the MOF structures and adsorption properties, a set of 20 MOFs was sampled from this collection for more detailed calculations. The identities of these MOFs and some of their physical properties are listed in Table S1. We used the algorithm of Lei et al.^40^ to ensure a range of chemical diversity in the subset of MOFs for this purpose. For each of these 20 MOFs, we determined the MPDC as described above using DFT-relaxed structures for each defect concentration we considered. The resulting MPDC values are shown in Figure 3. Except for one material for which the MPDC is 0.25, all of the other examples have an MPDC less than 0.12. For MOFs with mixed linkers, defective structures with different missing linker types have slightly different tolerances for defects, while the MPDC is defined as the maximum value among defective structures. Four examples of this outcome are shown in Figures S1–S4. Two of the MOFs with missing nitrogen-coordinated linkers have a higher MPDC compared to materials with oxygen-coordinated linkers, but two other MOFs with nitrogen-coordinated linkers fall in the same range as the oxygen-coordinated materials.

**Figure 3:**
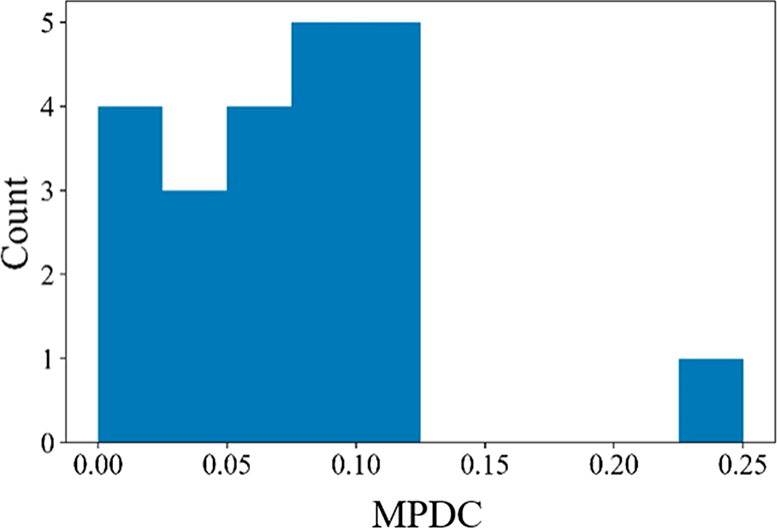
Histogram of MPDC for 20 chemically diverse MOFs selected as described in the text.

It is interesting to consider whether there are any readily identifiable characteristics that correlate with high values of the MPDC. The data in Figure 2, especially for UiO-66 and ZIF-8, suggest that the MPDC might correlate to the coordination number of metal clusters in a MOF. As shown in Figure 4a, however, among the larger set of materials we examined, there is not a strong correlation between coordination number and MPDC. Similarly, it may seem intuitive that defect-free MOFs with high mechanical stability would be associated with higher values of the MPDC. We examined this idea by computing the Young’s modulus and shear modulus for each MOF predicted using MEGNet (see Figure 4b and the Supporting Information for more details). When the mechanical moduli for each material were computed, we found no evidence of a correlation between either modulus and the materials’ MPDC.

**Figure 4:**
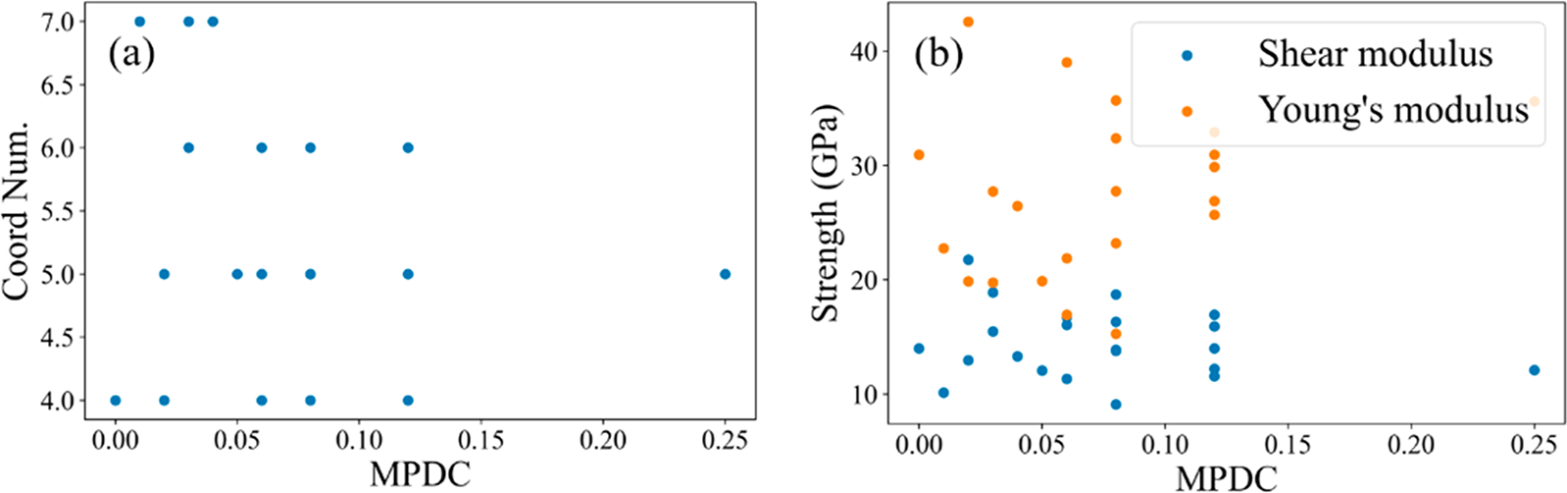
Relationship between MPDC for the 20 selected MOFs and (a) the coordination number for the metal center. (b) Mechanical properties.

The availability of a wide range of defective MOF structures allows us to consider how the presence and concentration of defects impact molecular adsorption. We performed Grand Canonical Monte Carlo (GCMC) simulations of molecular adsorption in a range of materials (Supporting Information). Figure S5 shows an example of these calculations for single-component adsorption of ethane, ethene, CO_2_, and H_2_S in UiO-66 as a function of defect concentration. For each molecule, the defect concentration has only a small impact on the adsorbed loading at low loadings, but at higher loadings, the presence of defects leads to substantial changes in the adsorbed amount. The trend of CO_2_ adsorption isotherm from our study agrees with the previous studies of Wu et al.^24^ and Jajko et al.^41^ Further discussion of the data for UiO-66 can be found in the SI. The simulated adsorption isotherms of ethane, ethene, CO_2_, and H_2_S for HKUST-1, IRMOF-1, and ZIF-8 are shown in Figures S6–S8. In all of these cases, defects do not have a significant influence on the adsorption. Below, we discuss the trends observed for molecular adsorption among the 20 randomly selected MOFs mentioned above. Throughout this discussion, we considered adsorption at room temperature and only reported results for MOFs with defect concentrations lower than their MPDC. We report adsorption at a series of pressures for each molecule, and in each case, the lowest pressure gives information about dilute loadings that can be considered to be in the Henry’s limit.

We used ethane and ethene as prototypical small nonpolar organic molecules. In general, these molecules preferentially adsorb near organic linkers and do not show strong binding to OH or H_2_O terminated defects. These two adsorbates show similar trends among the various materials, so the adsorption results for ethene are shown in Figure 5 while the results for ethane are shown in Figure S10. Figure 5a shows the normalized adsorption loading relative to the defect-free material as a function of pressure averaged across the set of materials as a function of pressure and defect concentration. For visualization purposes, the data are shown for the closest available defect concentration to 0.02, 0.05, 0.08, and 0.10. Because few materials have MPDC > 0.10, data with defect concentrations higher than 0.10 were not included in the plot. The variation of this normalized loading among the set of MOFs at two specific defect concentrations is shown in Figure 5b and c.

**Figure 5:**
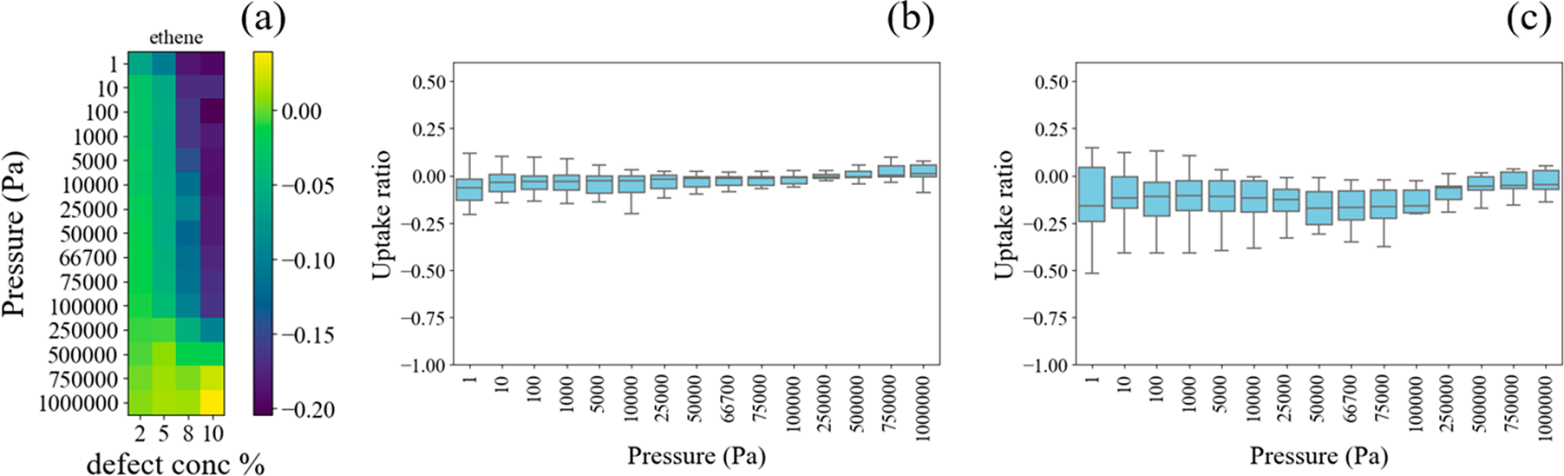
Normalized ethane loading in 20 defective MOFs at room temperature. Each loading is normalized to the loading in the associated defect-free structure at the same pressure and is displayed using a log scale. (a) Normalized isotherms averaged over 20 MOFs; color bar represents the log_10_ (uptake ratio); (b) uptake ratio distribution of 20 MOFs at 2.5 defect concentration. (c) Uptake ratio distribution of 20 MOFs at a defect concentration of 10 defect concentration.

As shown in Figure 5a and b, the log scale loading ratio for ethene for structures at low defect concentrations (e.g., 0.02, 0.05) has a mean value around zero and is distributed between 0 and −0.1. This means that these low defect concentrations change the adsorption of ethene by a small amount, consistent with the expectation that ethane and ethene do not have a strong affinity for the metal clusters or the OH/H_2_O groups. For defective structures at higher defect concentrations (i.e., 0.08, 0.1), the mean log scale loading ratio is around −0.15 at pressures below 1 bar, with values distributed between 0 and −0.25 (see Figure 5a and c). This means that in these conditions most MOFs have on average 30% lower molecular loading than defect-free materials. This is consistent with the expectation that there are fewer adsorption sites near organic linkers as the defect concentration increases.

At high pressures, the difference between ethene or ethane adsorption in the defect-free structure and defective structures becomes smaller because the adsorbates tend to fill all of the empty space within the MOFs, and the loading is determined by the pore size. However, we can see from Figure 5b and c, there are some outliers where defects have a positive influence on the ethene uptake. Those are MOFs whose pore size is close to the kinetic diameter of ethane/ethene (∼4.4 Å), in which case the missing linkers help to enlarge the pore size to allow the adsorbates to fit into the pores.

CO_2_ has a nonzero quadrupole moment, and its adsorption mechanism differs from that observed for nonpolar molecules. Multiple previous studies show that coordinated water molecules near defect sites can affect CO_2_ adsorption in MOFs.^42^ For example, Hardian et al. studied MOF-808 and found that the CO_2_ uptake is slightly higher in defective structures.^10^ In the work of Erucar and Keskin, compared to defect-free MOF, drastic decreases in CO_2_ working capacities are observed for CO_2_/N_2_ separation in a defective MOF.^43^ Yu and Balbuena found a similar trend for CO_2_ adsorption in MOF-74, where water coordinated near open metal sites resulted in a weaker interaction between CO_2_ and the MOF.^44^

We simulated the adsorption of CO_2_ in 20 MOFs as a function of the defect concentration to explore the trends in adsorption. As shown in Figure 6a and b, the log scale loading ratio of CO_2_ adsorption at low pressure and low defect concentrations has a mean value around zero and is distributed in a small range from 0.03 to −0.5. That is, although there are several cases where defects have a slightly positive influence, defects typically have a negligible influence or a slightly negative influence on the CO_2_ adsorption in these cases. This is reasonable because we cap the defect sites with OH/H_2_O, which does not introduce a strong binding site for CO_2_ in most MOFs. Compared to ethene and ethane, the magnitude of the influence of defects is slightly higher for CO_2_ because CO_2_ is more sensitive to changes in the local environment due to the additional contributions from Coulombic interactions.

**Figure 6:**
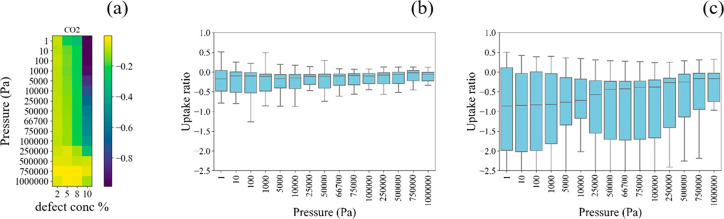
Normalized CO_2_ uptake in 20 defective MOFs at room temperature. The uptake is normalized to the associated defect-free structure and uses a log scale. (a) Normalized isotherms averaged over 20 MOFs; color bar represents the log_10_ (uptake ratio); (b) uptake ratio distribution of 20 MOFs at 2.5 defect concentration; (c) uptake ratio distribution of 20 MOFs at a defect concentration of 10 defect.

For structures with high defect concentrations (i.e., 0.1), defects negatively influence the adsorption of CO_2_. We note that there are only five MOFs that have an MPDC larger than 0.1, so the number of MOFs analyzed for Figure 6b and c are different. The distribution in Figure 6c is wide, which means that the influence of defects varies considerably among the limited number of MOFs that support a defect concentration of 0.1. Notably, there are materials in which the defects increase the level of CO_2_ adsorption but other materials in which the level of CO_2_ adsorption is strongly diminished due to defects. The variation in the influence of defects on CO_2_ adsorption becomes narrower at high pressure, because the pore size is the dominant factor in the adsorption loading under these conditions.

We studied the influence of defects on methanol adsorption as methanol is often involved in the CO_2_ hydrogenation process in MOFs.^45^ The reproducibility of experimental methanol adsorption isotherms in MOFs has been analyzed previously by Bingel et al.^46^ Unlike CO_2_ and ethane/ethene, small concentrations of defects have a significant positive influence on the uptake of methanol, especially at low pressures (see Figure 7). As shown in Figure 7b, the distribution of the log scale influence of defects on methanol adsorption spans from 0 to 4. This occurs because H_2_O/OH-capped defect sites create strong binding sites for methanol via the formation of hydrogen bonds. It is interesting to note that simulations based on defect-free structures by Bingel et al. underestimated the methanol loading at low activities relative to consensus experimental isotherms developed from multiple independent measurements in most but not all MOFs.^46^ This may be because of the effects of small concentrations of defects on the experimentally measured materials. It is important to note that all of the MOFs reported in Figure 7 had defect concentrations lower than their MPDC, which means that routine experimental characterization of these materials would not have identified them as differing noticeably from a defect-free MOF structure. In other words, any of the adsorption results from Figure 7 would have been reported without any indication of the importance of defects if the data were obtained experimentally. As with the other molecules discussed above, the impact of defects on the adsorbed loadings of methanol diminishes at higher pressures.

**Figure 7:**
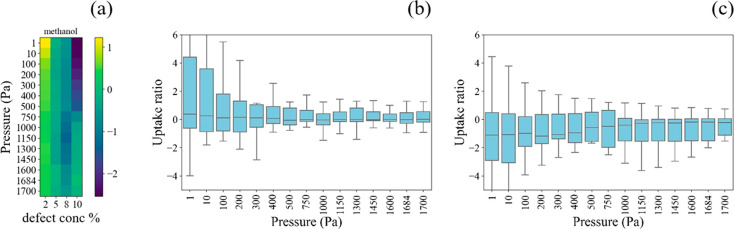
Normalized methanol uptake in 20 defective MOFs at room temperature. The uptake is normalized to the associated defect-free structure and uses a log scale. (a) Normalized isotherms averaged over 20 MOFs; color bar represents the log_10_ (uptake ratio); (b) uptake ratio distribution of 20 MOFs at 2.5 defect concentration; (c) uptake ratio distribution of 20 MOFs at defect concentration of 10.

In conclusion, defects are ubiquitous in MOFs, but until now, defective MOF structures could not be generated easily, which limited high-throughput studies to structures that are defect-free. We have introduced a tool that can easily generate missing-linker defective MOF structures in a high-throughput way. We validated our tool by comparing our results to the literature, using four MOFs that have been widely synthesized. To demonstrate the effectiveness of our approach, we generated a collection of defective structures for 507 MOFs. The tools we developed and the sample structures are available as described in the Supporting Information.

We chose 20 MOFs and relaxed their defective structures at multiple defect concentrations using DFT to allow for more detailed analysis. We introduced the concept of the maximum possible defect concentration (MPDC) to estimate a defect concentration above which deviations from results based on the defect-free structure in commonly reported experimental quantities would readily be observed. A simple rule was used to define the MPDC based on PXRD pattern similarity and surface area change compared with the defect-free structures, but we acknowledge that refinements of this definition may be needed in some cases. For the 20 randomly selected MOFs we examined, 19 have an MPDC smaller than 0.12. For MOFs with mixed linkers, the missing linker types do not have a strong influence on the MPDC. Although the metal coordination number and mechanical properties may intuitively appear to be correlated with the MPDC, we found that neither of these quantities had any predictive power. One interesting direction for future work may be to develop physical descriptors that are well correlated with the MPDC.

The influence of defects on molecular adsorption depends on the defect concentration, capping agent, adsorbate, and pressure. We performed GCMC simulations of single-component adsorption of ethene, ethane, CO_2_, and methanol as prototypical nonpolar and polar adsorbates. We only used MOFs at defect concentrations lower than the MPDC, meaning that all of the defective MOFs in these GCMC simulations would have been considered as well-defined crystalline structures in typical experimental studies of MOFs. At low pressures, defects have a slightly negative or negligible influence on adsorption if they do not introduce selective binding sites for adsorbed molecules. Ethene, ethane, and CO_2_ all fall into this category as we used OH^−^/H_2_O as the capping agents for point defects. Defects have a strong positive influence on methanol adsorption at low pressure, however, as the capping agents can form hydrogen bonds with the adsorbing molecules. At high pore loadings, the adsorption loadings are mainly dictated by the pore volume. Thus, defective structures tend to have a slightly higher uptake than defect-free structures for all adsorbates at high pressures.

## Supporting Information Available

Mechanical properties for 20 randomly selected MOFs, tabulated data for adsorption isotherms for four adsorbates in 20 MOFs at defective and defect-free structures, DFT relaxation convergence details (ZIP)
Computational methods, correlation between MPDC and missing linker type, benchmark using widely synthesized MOFs, MEGNet benchmark, list of 20 randomly selected MOFs, and additional adsorption isotherms (PDF)
Transparent Peer Review report available (PDF)
