# Supplementary material for: Efficient Generation of Large Collections of Metal–Organic Framework Structures Containing Well-Defined Point Defects
Source: J Phys Chem Lett. 2023 Jul 18;14(29):6658–65. doi: 10.1021/acs.jpclett.3c01524 (PMC10388356; doi:10.1021/acs.jpclett.3c01524)
Supplement: Supplementary file 2 — jz3c01524_si_002.pdf [file jz3c01524_si_002.pdf]

Supporting information

Efficient Generation of Large Collections of Metal-Organic Framework Structures Containing Well-Defined Point Defects

Zhenzi Yu,[1] Shubham Jamdade,[1] Xiaohan Yu,[1] Xuqing Cai,[1] and David S. Sholl[1,2]*

[1] School of Chemical & Biomolecular Engineering, Georgia Institute of Technology, Atlanta, Georgia, 30332, USA

[2] Oak Ridge National Laboratory, Oak Ridge, Tennessee, 37830, USA

Corresponding Author

*E-mail: shollds@ornl.gov. Phone: 1-4048942822 (D.S.S.).

Contents

Methods 2
Software implementation 2
Structure optimization and charge assignment 2
GCMC Simulations 2
Characterization 3
Data availability 3
Correlation between MPDC and missing linker type 3
Benchmark using widely studied MOFs 5
UiO66 5
HKUST-1 6
IRMOF-1 7
ZIF-8 7
Influence of defects on Ethene adsorption 8
Influence of defects on Ethane adsorption 9
Influence of defects on CO_2 adsorption 10
Influence of defects on methanol adsorption 11
MEGNet benchmarking 11
20 randomly selected MOFs 12
Reference 13

Methods

Software implementation

We developed a Python repository that can generate missing linker defect structures with different missing linker types and defect concentrations for arbitrary MOF crystal structures (https://github.com/zyu331/mof_defect_builder.git). The pipeline consists of three parts: identification of linkers and nodes, capping of OMS, and SRO adjustment. The identification of linkers and nodes is based on the MOF_ID work by Bucior et al.[1] After the linkers are identified, the charge is assigned using the GasteigerCharge method from RDKit. We found that in a small number of cases, RDKit cannot assign charges due to internal errors, and these cases were resolved manually.

Our algorithm for OMS capping was modified from the vector-sum method of Rosen et al, which analyses the coordination environment near the metal cluster and then defines the geometrical positions of capping agents.[2] As already mentioned, due to computational limits we do not consider examples that explore the influence of SRO on defects in this paper. In all the examples below we randomly removed linkers from the structure with the constraint that linker removal does not create covalently bonded fragments that are not connected by any bonds with the extended MOF structure. Nevertheless, the reverse Monte Carlo algorithm of Verploegh et al.[3] that allows control of SRO via the Warren-Cowley parameter is implemented in our code and can be used to generate defective structures with varying SRO. As an illustration of the generality of this approach, we created a missing-linker MOFs defect structure database for 507 MOFs that had previously been optimized using DFT from the larger collection of CoRE MOFs.[4] More technical details and the database can be found in the open-source GitHub repository.

Structure optimization and charge assignment

MOF structure relaxations were calculated using spatially periodic DFT in the Vienna ab initio simulation package,[5] along with a plane-wave basis set and projected-augmented wave[6] pseudo-potentials. All calculations used the Perdew, Burke, and Ernzerhof generalized gradient approximation exchange–correlation functional[7] with D3 dispersion corrections (PBE-D3).[8] We simultaneously optimized both the lattice parameters and atomic positions using plane-wave cutoff energy of 600 eV and Γ-point sampling for Brillouin zone integration. Using a quasi-Newton method, we originally relaxed geometries until the force on each atom was smaller than 0.05 eV/Å. The point charges on atoms of the resulting structure were assigned using the DDEC6 method.[9]

From the perspective of DFT calculations, the unit cells needed for low defect concentrations are often large, leading to slow convergence. If a structure of a MOF with a higher defect concentration converged in our DFT calculations without significant changes in the simulated PXRD pattern and surface area, we loosened the convergence criteria for the structure(s) with a lower defect concentration. The optimized structures and associated convergence criteria are supplied in the SI.

GCMC Simulations

Grand Canonical Monte Carlo (GCMC) simulations were used to simulate room-temperature single-component adsorption. The simulations utilized the RASPA software [10,11] and employed the TraPPE force field [12,13] to describe the van der Waals (vdW) interactions between adsorbates. Adsorbate-MOF interactions were defined using Lorentz-Berthelot mixing rules, with the vdW parameters for MOF atoms obtained from the UFF4MOF.[14,15] Lennard-Jones interactions were truncated at 12 Å and Coulombic interactions were modeled using the long-range Ewald summation scheme with a relative accuracy of 10^{-6}. MOF unit cells were replicated to a minimum of 24 Å along each dimension under triclinic periodic

boundary conditions in all dimensions. Monte Carlo trial moves including translation, rotation, reinsertion, deletion, and insertion moves were attempted with equal probabilities during GCMC. The simulations used 10^5 equilibration and 10^5 production cycles, which were shown to yield well-converged results in initial tests.

Characterization

We used the MEGNet, a graph neutral network force field (FF) trained on 60,000 crystals from Material Project, to examine the mechanical properties of defective MOFs.[16] We used MEGNet because it is easy to implement compared to traditional FFs, which are not necessarily straightforward to define for defective MOFs, and much quicker than first-principles methods. MEGNet aims to provide a DFT level of accuracy, but it has not been benchmarked for crystals containing defects. A comparison of mechanical properties predicted by MEGNet and UFF4MOF is illustrated in Figure S13. Bulk Modulus prediction from these two levels of theory agrees with each other qualitatively, while the shear modulus differs. According to the previous study, we argue MEGNet provides a better approximation to the DFT level of accuracy.[17,18] Specific simulation procedures can be found in the GitHub document associated with the Jupyter Notebook. (https://github.com/materialsvirtuallab/megnet)

Pymatgen was used to simulate the powder XRD pattern of selected MOFs.[19] The Scherrer equation is used to broaden the peak by assuming a mean crystal size of 10 μm. Many examples of MOF synthesis give crystals that are smaller than this, which would be associated with sharper PXRD peaks. MOF surface areas were calculated using Zeo++.[20] These calculations used a probe diameter of 1.32 Å to mimic Helium.

Many metrics have been proposed to quantitatively compare PXRD spectra.[21] We calculated PXRD similarity scores using the second-order Minkowski metric, which is defined as

$$1 - \frac{\sum(PXRD_1 - PXRD_2)^2}{\sum PXRD_1^2 + \sum PXRD_2^2}$$

where $PXRD_1$ and $PXRD_2$ are the normalized PXRD intensities of the defect-free structure and a defective structure, respectively. PXRD patterns in calculating this similarity score are normalized to their highest peak. This similarity score gives values in the range [0,1], where 1 means the two PXRD patterns are identical.

Data availability

The data used to make the figures in this paper are all included in the ZIP file in the SI. The CO_2/methanol adsorption isotherms are in the Adsorption_CO2_methanol.xlsx, while ethene/ethane adsorption sioterhms are in Adsorption_ethene_ethane.xlsx. The Mechanical proprerties calculated by FF and MEGNet are in the Mechenical_properties.xlsx. The DFT convergency information and physical proprerties are in the 20_random_MOFs.xlsx.

The generated defective structure database for MOFs and example input for simulation can be found in the online Github repository mentioned above.

Correlation between MPDC and missing linker type

We have attached the PXRD pattern for MOFs with different missing linker types. As we can see from Figures S1 and S4, the identified MPDC does not have a strong difference for the two different missing

linker types. On the contrary, the identified MPDC has slight differences in two MOF examples as shown in Figures S2 and S3.

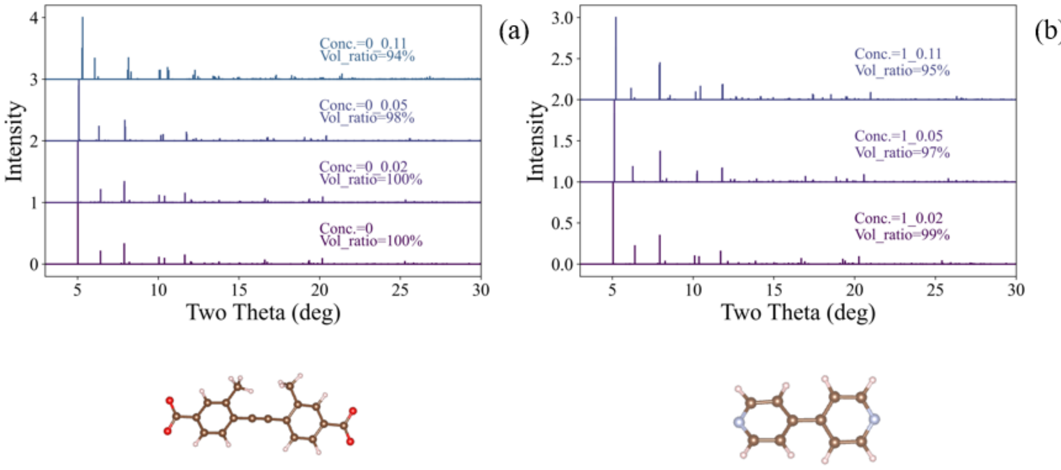

Figure S1 PXRD pattern and surface area of AFITIT with two types of missing linkers: (a) C16H12O42-, (b) C10H8N2. The associated missing linker is shown below the PXRD pattern.

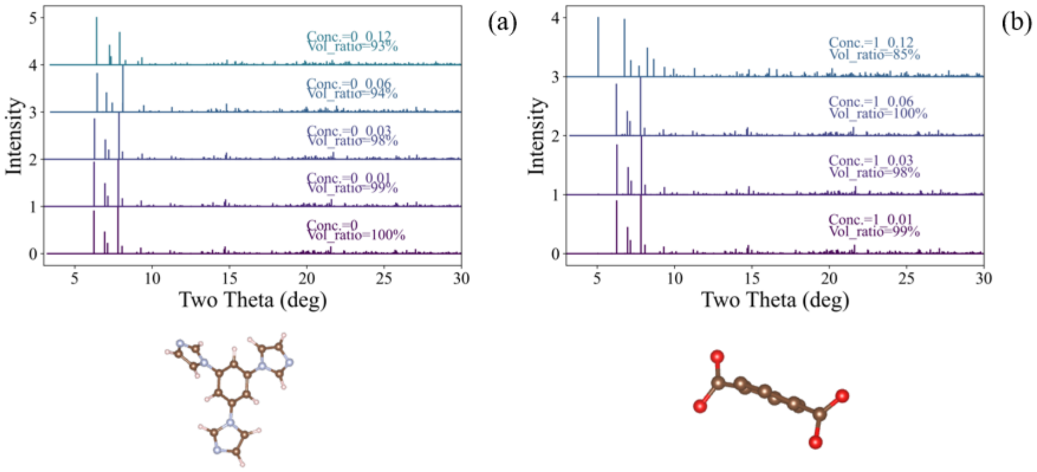

Figure S2 PXRD pattern and surface area of AFITIT with two types of missing linkers: (a) $C_{16}H_{12}O4^{2-}$, (b) $C_{10}H_8N_2$. The associated missing linker is shown below the PXRD pattern.

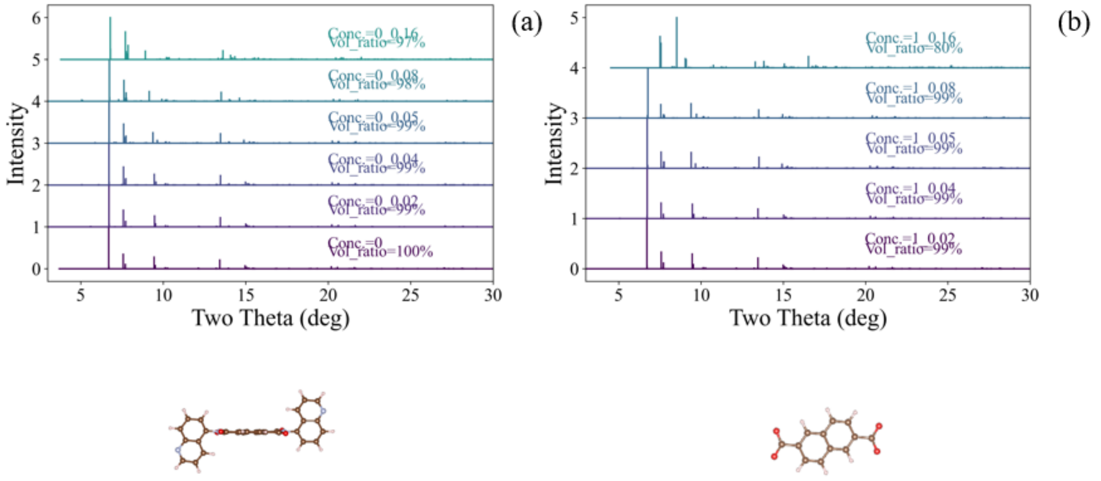

Figure S3 PXRD pattern and surface area of COGWEB with two types of missing linkers: (a) $C_{26}H_{16}O_4N_6$, (b) $C_{12}O_4Br_6^{2-}$. The associated missing linker is shown below the PXRD pattern.

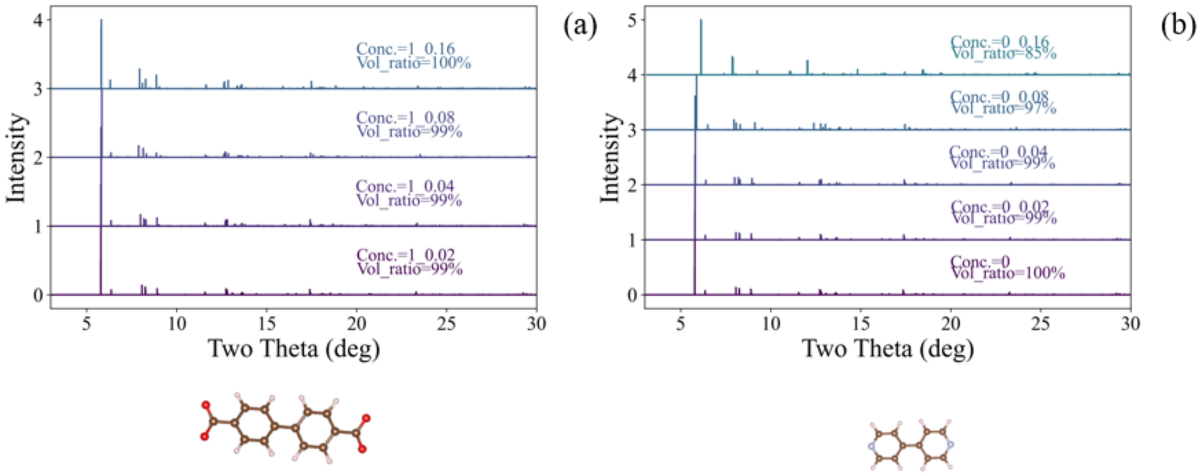

Figure S4 PXRD pattern and surface area of FEFCUQ with two types of missing linkers: (a) $C_{14}H_8O_4^{2-}$, (b) $C_{10}H_8N_2$. The associated missing linker is shown below the PXRD pattern.

Benchmark using widely studied MOFs

UiO66

As shown in Figure 5 (a) and (b), when pressure is below 1 bar, there is no significant uptake difference among all structures. The defect-free structures at high concentrations have a slightly lower uptake. Because the missing linkers reduce the VDW interactions between adsorbates and the framework, while ethane/ethene has no affinity for the metal clusters or the OH/H_2O groups. At higher pressure, the uptake is higher in structures with higher defective concentration, because the adsorbates start to fill all space within the MOFs, and the uptake amount is determined by the pore size.

CO_2 and H_2S both have nonzero quadrupole moment, and their adsorption mechanism differs from that observed for non-polar molecules. As shown in Figures 5 (c) and (d), defects do not affect the shape of

the adsorption isotherm. Below 1 bar, there is also no significant difference among all structures. CO_2 is mostly absorbed near the organic linkers, avoiding the metal clusters containing OH/H_2O groups (which are the preferential adsorption sites for molecules that can form hydrogen bonds). Thus, the defects do not introduce new binding sites for CO_2 at low pressure. At higher pressure, we observe that structures with moderate defect concentrations have the highest uptake. This is because of the tradeoff between pore volume increasing and the VDW interaction between organic linkers and CO_2 decreasing. Note that there is a difference between our work and the work of Jajko et al.[22], which might be contributed to the different capping agent choices and SRO settings. (We have a random defect distribution, and their structure has a clustered distribution)

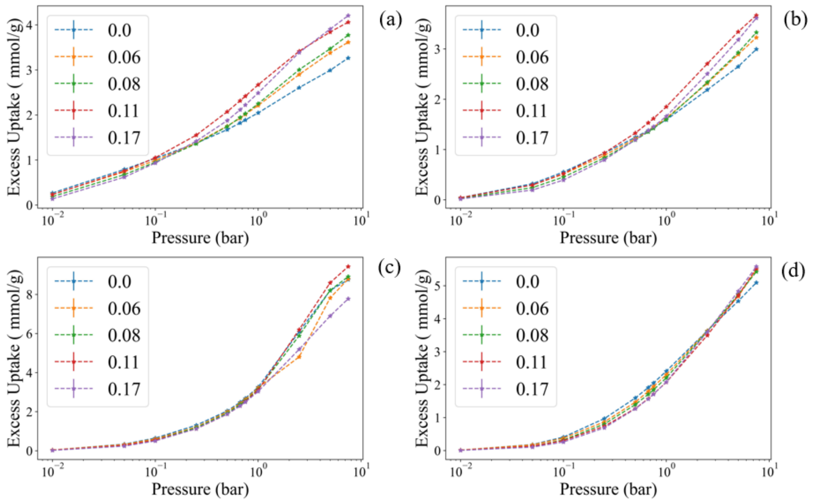

Figure S1 Adsorption isotherms for (a) ethane (b) ethene (c) CO_2 (d) H_2S in UiO-66 with different defect concentrations at 298 K. The lines with different colors represent defective structures with different defect concentrations.

HKUST-1

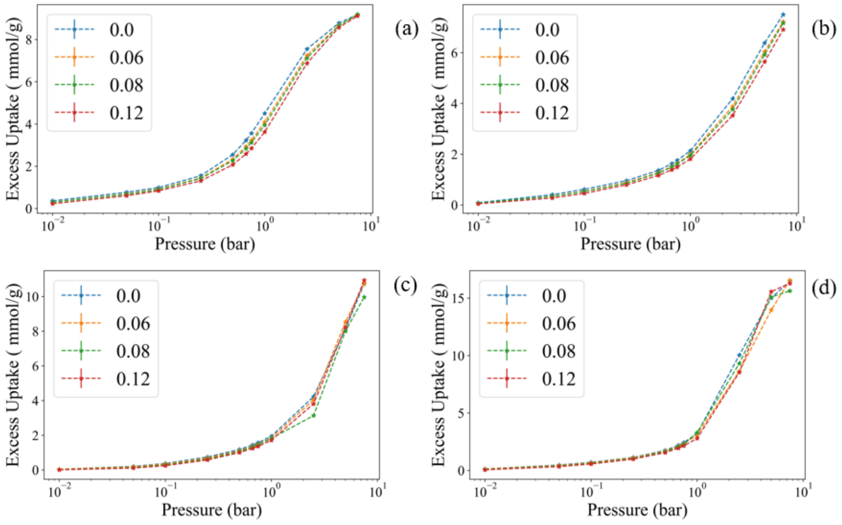

Figure S5 Adsorption isotherm for (a) ethane (b) ethene (c) CO_2 (d) H_2S in HKUST-1 with different defect concentrations at 298 K. The lines with different colors represent defective structures with different defect concentrations.

IRMOF-1

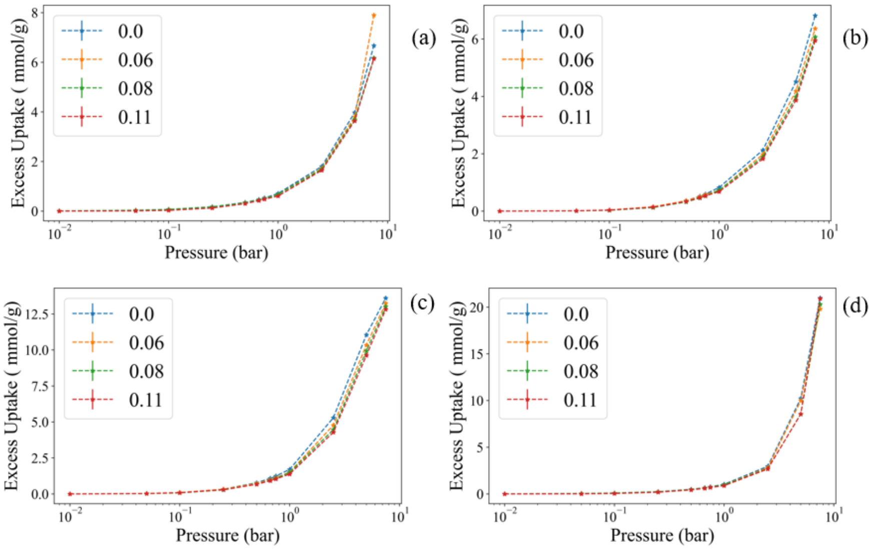

Figure S6 Adsorption isotherm for (a) ethane (b) ethene (c) CO_2 (d) H_2S in IRMOF-1 with different defect concentrations at 298 K. The lines with different colors represent defective structures with different defect concentrations.

ZIF-8

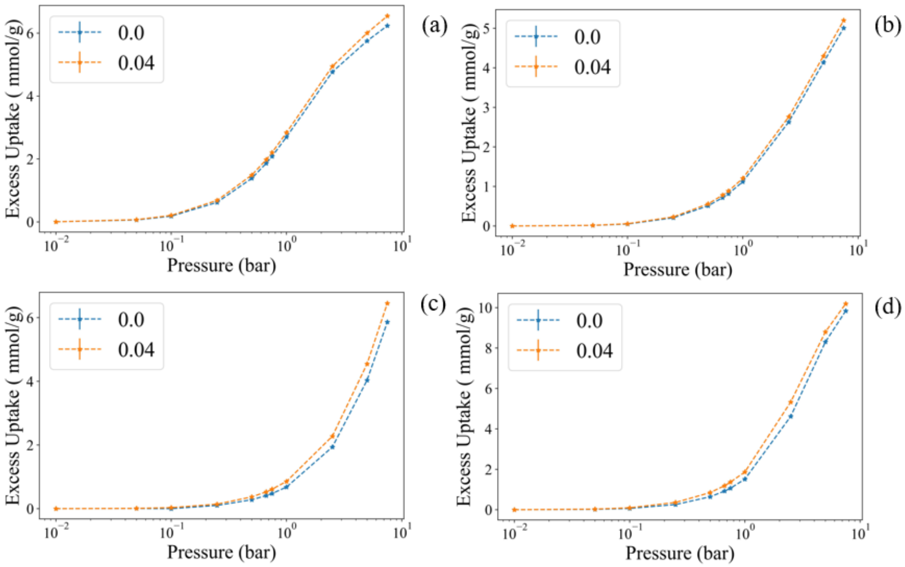

Figure S7 Adsorption isotherm for (a) ethane (b) ethene (c) CO_2 (d) H_2S in ZIF-8 with different defect concentrations at 298 K. The lines with different colors represent defective structures with different defect concentrations.

Influence of defects on Ethene adsorption

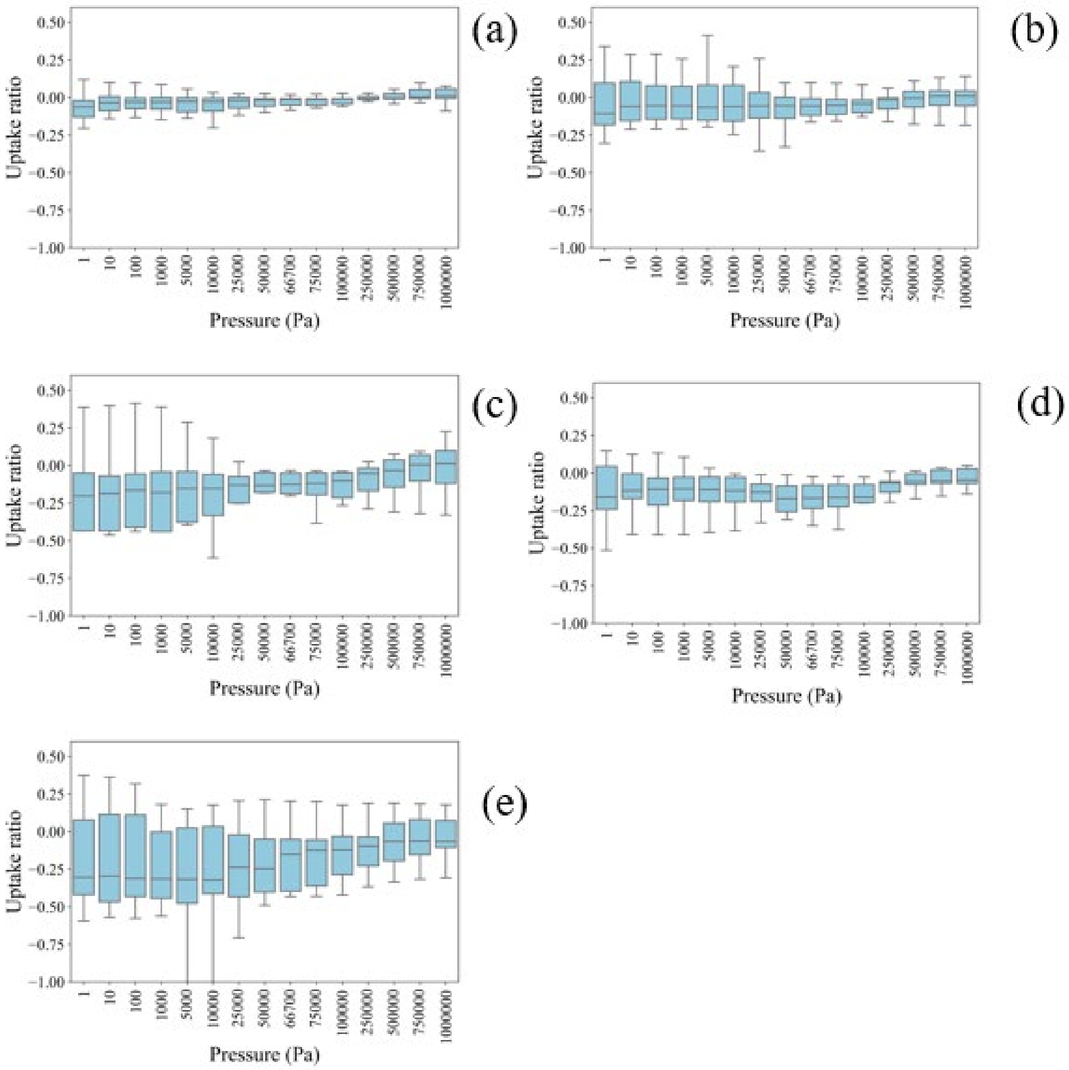

Figure S8 Normalized ethene uptake distribution in 20 defective MOFs at 298 K. The uptake is normalized to the associated defect-free structure and uses a log scale. (a) in structures with a defect concentration of 0.02 (b) in structures with a defect concentration of 0.05 (c) in structures with a defect concentration of 0.08 (d) in structures with a defection concentration of 0.1, (e) in structures with a defect concentration of 0.12. All structures are under their MPDC and DFT optimized.

Influence of defects on Ethane adsorption

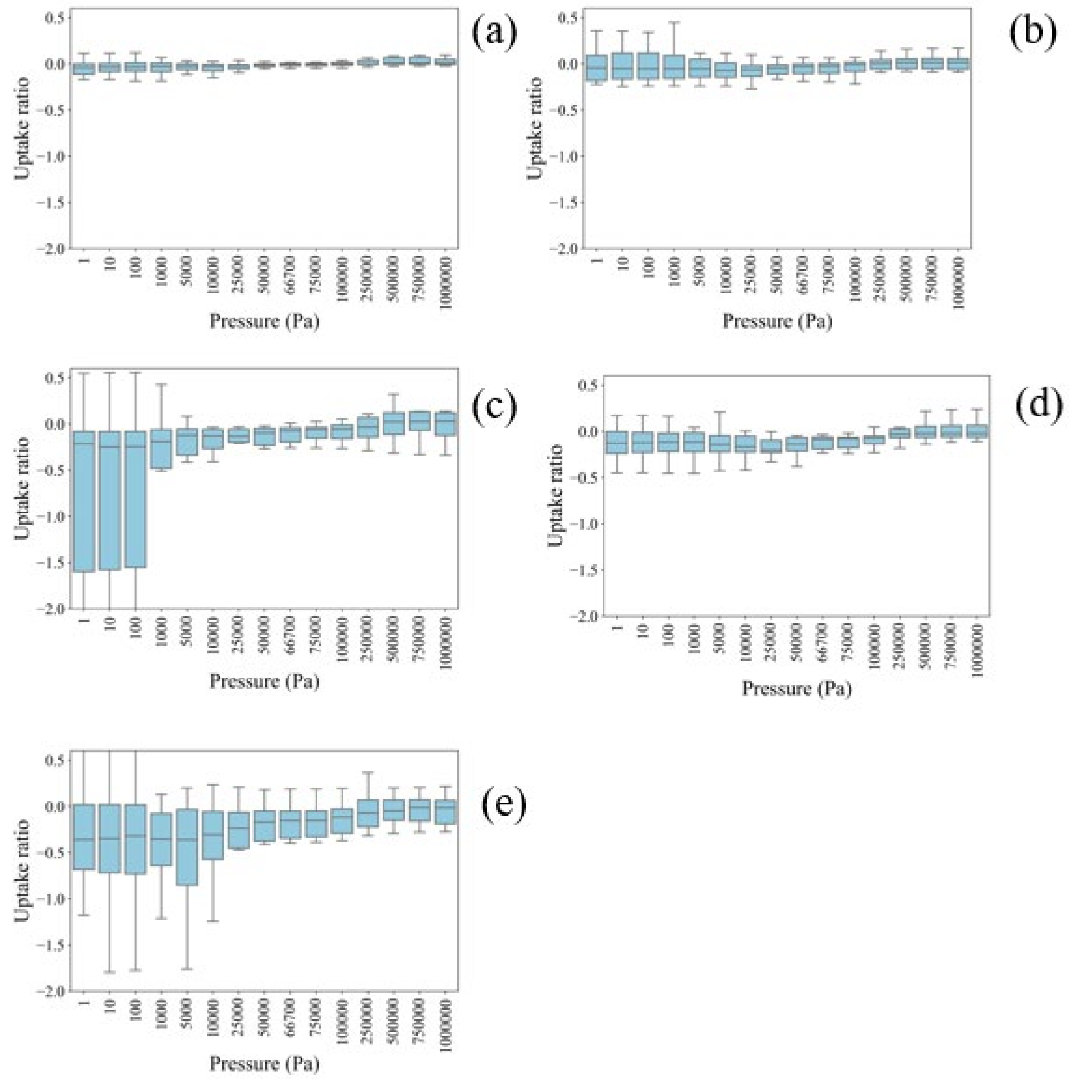

Figure S9 Normalized ethane uptake distribution in 20 defective MOFs at 298 K. The uptake is normalized to the associated defect-free structure and uses a log scale. (a) in structures with a defect concentration of 0.02 (b) in structures with a defect concentration of 0.05 (c) in structures with a defect concentration of 0.08 (d) in structures with a defect concentration of 0.1, (e) in structures with a defect concentration of 0.12. All structures are under their MPDC and DFT optimized.

Influence of defects on CO_2 adsorption

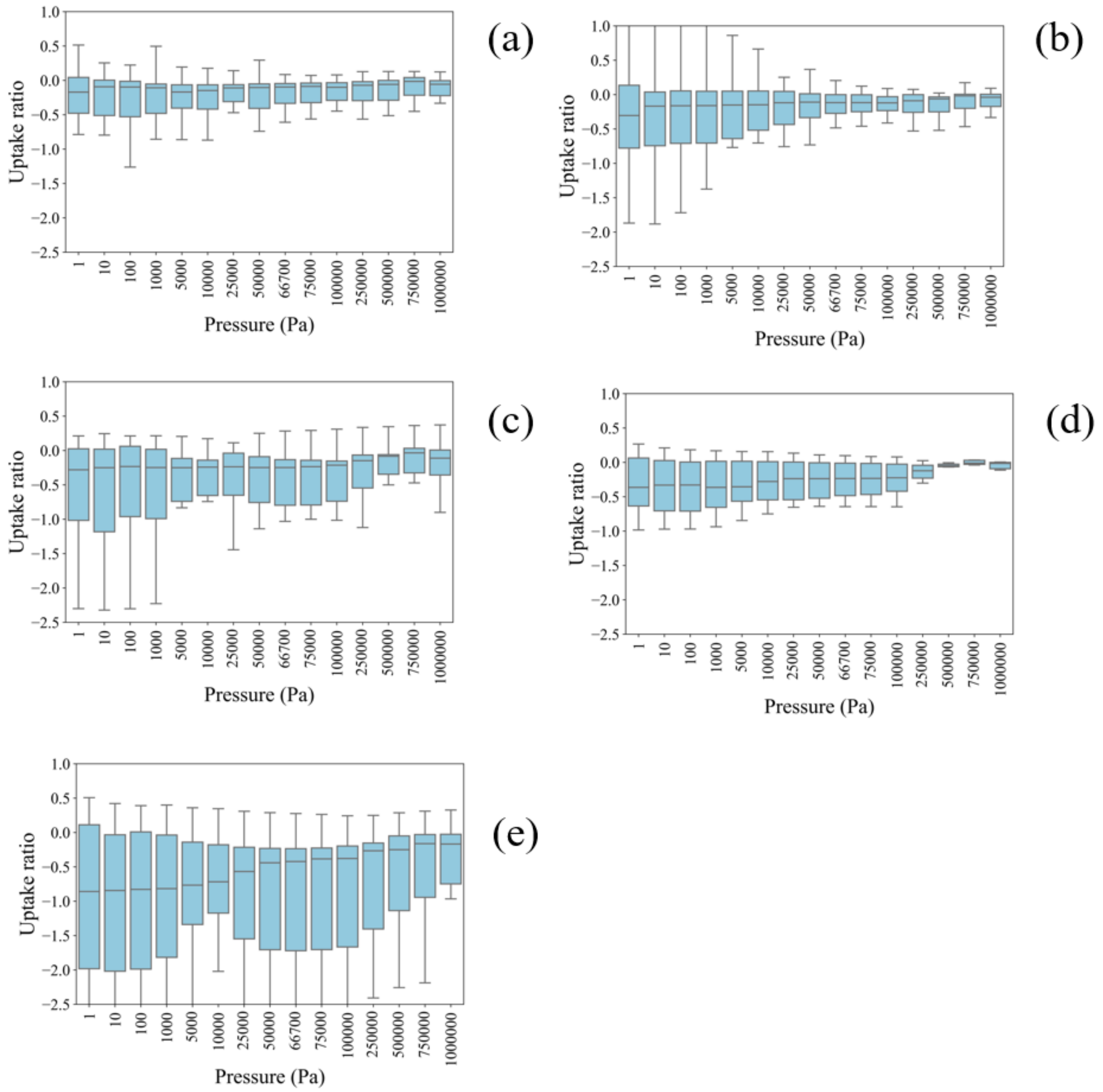

Figure S10 Normalized CO_2 uptake distribution in 20 defective MOFs at 298 K. The uptake is normalized to the associated defect-free structure and uses a log scale. (a) in structures with a defect concentration of 0.02 (b) in structures with a defect concentration of 0.05 (c) in structures with a defect concentration of 0.08 (d) in structures with a defect concentration of 0.1, (e) in structures with a defect concentration of 0.12. All structures are under their MPDC and DFT optimized.

Influence of defects on methanol adsorption

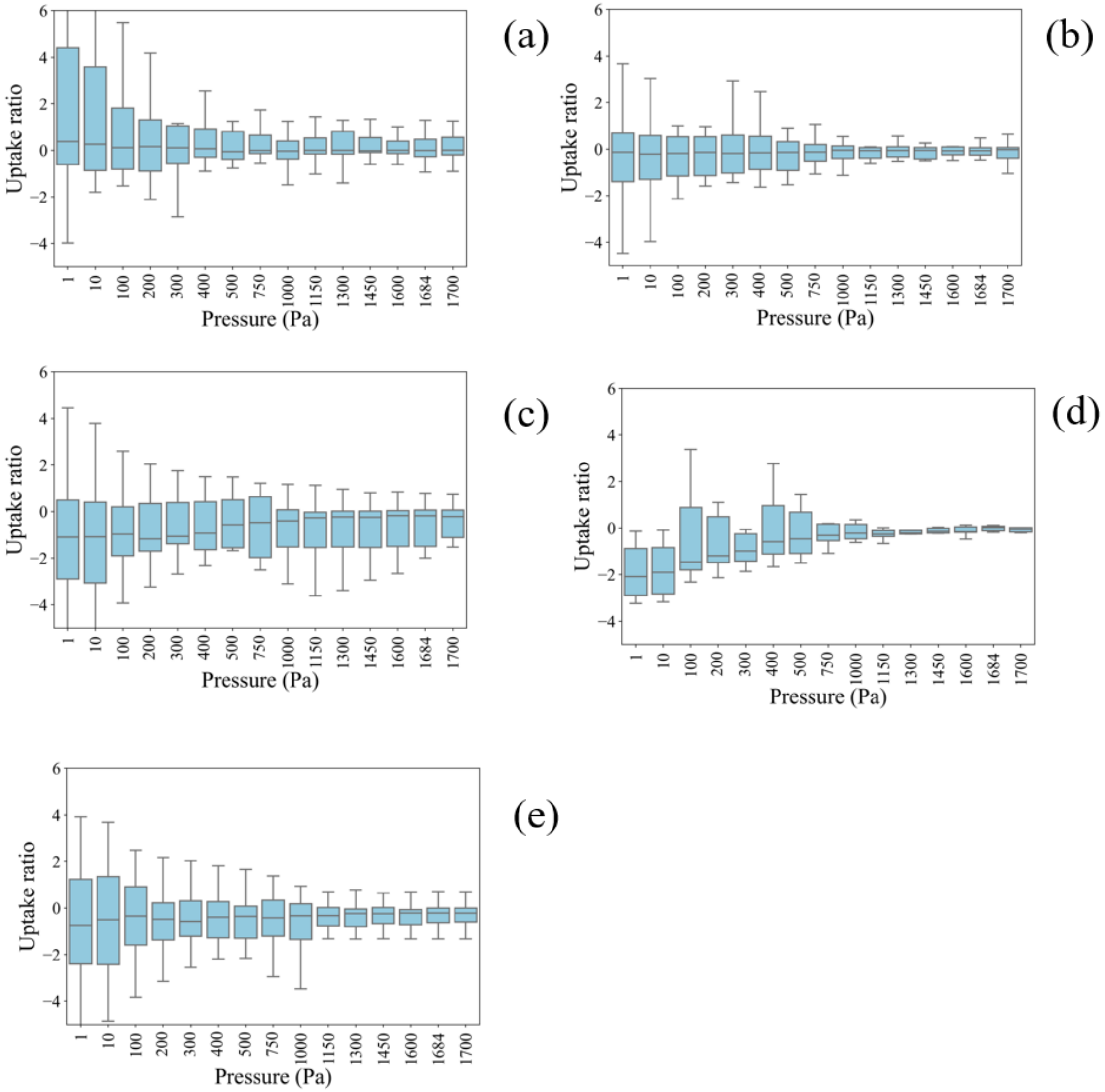

Figure S11 Normalized methanol uptake distribution in 20 defective MOFs at 298 K. The uptake is normalized to the associated defect-free structure and uses a log scale. (a) in structures with a defect concentration of 0.02 (b) in structures with a defect concentration of 0.05 (c) in structures with a defect concentration of 0.08 (d) in structures with a defect concentration of 0.1, (e) in structures with a defect concentration of 0.12. All structures are under their MPDC and DFT optimized.

MEGNet benchmarking

The mechanical property prediction by UFF4MOF is obtained using the method described in the work of Yu et al.[23] The simulation input can be found in the online GitHub repository. (https://github.com/zyu331/TAXI_MOF/tree/main/tools_ElasticConstant_MD) Defect-free structures of 20 randomly selected MOFs are used for the benchmark. As shown in Figure S13, the bulk modulus is correlated from two methods while the shear modulus is weakly correlated. The quantitative discrepancy can be attributed to the force field accuracy, extrapolation of the Graph Neutral network, etc. According to

the previous benchmark results, we anticipate that MEGNet provides a better approximation to the DFT level of accuracy.[18] Further investigation would be interesting but beyond the scope of this study. Specific simulation procedures can be found in the GitHub document: https://github.com/materialsvirtuallab/megnet

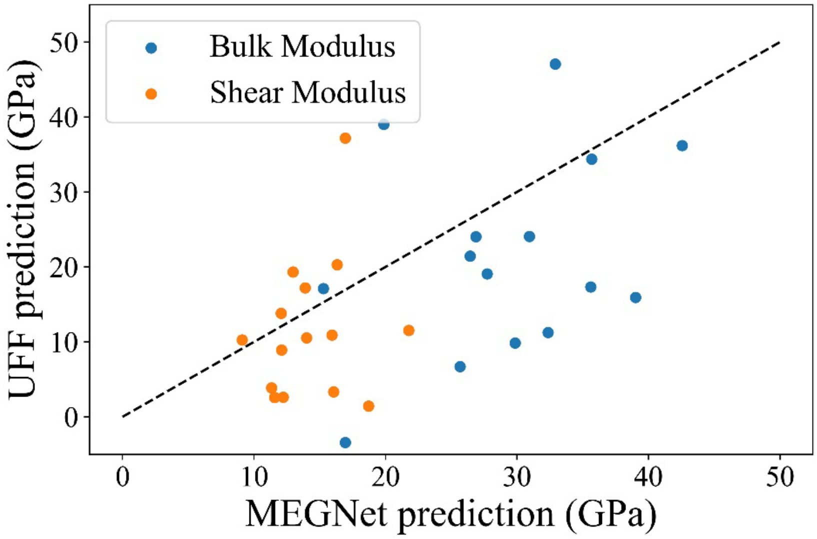

Figure S12 The mechanical properties comparison between UFF4MOF prediction and MEGNet prediction

20 randomly selected MOFs

Table S1 20 randomly selected MOFs and their physical properties

**MOF**	**LCD**	**PLD**	**Density**	**Accessible Surface Area**	**MOF**	**LCD**	**PLD**	**Density**	**Accessible Surface Area**
	**(Å)**	**(Å)**	**(cm^3/g)**	**(m^2/g)**		**(Å)**	**(Å)**	**(cm^3/g)**	**(m^2/g)**
KIYMAI	3.95	2.96	1.32	0	GUXLIU	3.77	2.4	1.89	0
COGWEB	6.37	5.7	0.92	2082	IXODUV	6.95	6.49	1.01	1764
BUVXOG	6.83	5.21	1.27	613	FUNCAT	9.49	8.78	0.93	1584
KIXXOG	3.84	2.67	1.17	0	NUYWOU	4.73	2.51	1.29	0
GINLIA	6.39	4.6	0.88	2497	FIQCEN	13.19	6.66	0.88	2405
FEFCUQ	8.88	6.35	0.78	2726	AFITIT	7.35	5.37	1.02	1359
DEYLUQ	7.28	4.67	1.62	604	EKOPOK	7.62	7.3	1.24	1051
FUNBEW	11.16	9.19	0.67	3135	GAXWEJ	4.4	2.71	1.57	0
HIFTOG	7.96	4.14	1.17	1628	DEYVUA	5.36	4.43	0.96	2613
KAYBIX	5.69	5.49	1.21	1102	AROFET	8.29	5.16	0.86	2602
IBICON	10.1	9.94	1.22	839	ECIWUJ	3.25	2.62	1.49	0

References

(1) Bucior, B. J.; Rosen, A. S.; Haranczyk, M.; Yao, Z.; Ziebel, M. E.; Farha, O. K.; Hupp, J. T.; Siepmann, J. I.; Aspuru-Guzik, A.; Snurr, R. Q. Identification Schemes for Metal-Organic Frameworks To Enable Rapid Search and Cheminformatics Analysis. *Cryst Growth Des* **2019**, *19* (11), 6682–6697. https://doi.org/10.1021/ACS.CGD.9B01050

(2) Rosen, A. S.; Notestein, J. M.; Snurr, R. Q. Identifying Promising Metal–Organic Frameworks for Heterogeneous Catalysis via High-Throughput Periodic Density Functional Theory. *J Comput Chem* **2019**, *40* (12), 1305–1318. https://doi.org/10.1002/JCC.25787.

(3) Verploegh, R. J.; Wu, Y.; Boulfelfel, S. E.; Sholl, D. S. Quantitative Predictions of Molecular Diffusion in Binary Mixed-Linker Zeolitic Imidazolate Frameworks Using Molecular Simulations. *Journal of Physical Chemistry C* **2018**, *122* (10), 5627–5638. https://doi.org/10.1021/ACS.JPCC.8B00781

(4) Nazarian, D.; Camp, J. S.; Chung, Y. G.; Snurr, R. Q.; Sholl, D. S. Large-Scale Refinement of Metal-Organic Framework Structures Using Density Functional Theory. *Chemistry of Materials* **2017**, *29* (6), 2521–2528. https://doi.org/10.1021/ACS.CHEMMATER.6B04226

(5) Kresse, G.; Furthmüller, J. Efficient Iterative Schemes for *Ab Initio* Total-Energy Calculations Using a Plane-Wave Basis Set. *Phys Rev B* **1996**, *54* (16), 11169. https://doi.org/10.1103/PhysRevB.54.11169.

(6) Blöchl, P. E. Projector Augmented-Wave Method. *Phys Rev B* **1994**, *50* (24), 17953. https://doi.org/10.1103/PhysRevB.50.17953.

(7) Perdew, J. P.; Burke, K.; Ernzerhof, M. Generalized Gradient Approximation Made Simple. *Phys Rev Lett* **1996**, *77* (18), 3865. https://doi.org/10.1103/PhysRevLett.77.3865.

(8) Grimme, S.; Antony, J.; Ehrlich, S.; Krieg, H. A Consistent and Accurate Ab Initio Parametrization of Density Functional Dispersion Correction (DFT-D) for the 94 Elements H-Pu. *J Chem Phys* **2010**, *132* (15), 154104. https://doi.org/10.1063/1.3382344.

(9) Manz, T. A.; Limas, N. G. Introducing DDEC6 Atomic Population Analysis: Part 1. Charge Partitioning Theory and Methodology. *RSC Adv* **2016**, *6* (53), 47771–47801. https://doi.org/10.1039/C6RA04656H.

(10) Dubbeldam, D.; Torres-Knoop, A.; Walton, K. S. On the Inner Workings of Monte Carlo Codes. *Mol Simul* **2013**, *39* (14–15), 1253–1292. https://doi.org/10.1080/08927022.2013.819102

(11) Dubbeldam, D.; Calero, S.; Ellis, D. E.; Snurr, R. Q. RASPA: Molecular Simulation Software for Adsorption and Diffusion in Flexible Nanoporous Materials. **2015**, *42* (2), 81–101. https://doi.org/10.1080/08927022.2015.1010082.

(12) Wick, C. D.; Martin, M. G.; Siepmann, J. I. Transferable Potentials for Phase Equilibria. 4. United-Atom Description of Linear and Branched Alkenes and Alkylbenzenes. *Journal of Physical Chemistry B* **2000**, *104* (33), 8008–8016. https://doi.org/10.1021/JP001044X

(13) Potoff, J. J.; Siepmann, J. I. Vapor–Liquid Equilibria of Mixtures Containing Alkanes, Carbon Dioxide, and Nitrogen. *AIChE Journal* **2001**, *47* (7), 1676–1682. https://doi.org/10.1002/AIC.690470719.

(14) Rappé, A. K.; Casewit, C. J.; Colwell, K. S.; Goddard, W. A.; Skiff, W. M. UFF, a Full Periodic Table Force Field for Molecular Mechanics and Molecular Dynamics Simulations. *J Am Chem Soc* **1992**, *114* (25), 10024–10035. https://doi.org/10.1021/JA00051A040

(15) Addicoat, M. A.; Vankova, N.; Akter, I. F.; Heine, T. Extension of the Universal Force Field to Metal-Organic Frameworks. *J Chem Theory Comput* **2014**, *10* (2), 880–891. https://doi.org/10.1021/CT400952T.

(16) Chen, C.; Ye, W.; Zuo, Y.; Zheng, C.; Ong, S. P. Graph Networks as a Universal Machine Learning Framework for Molecules and Crystals. *Chemistry of Materials* **2019**, *31* (9), 3564–3572. https://doi.org/10.1021/ACS.CHEMMATER.9B01294

(17) Moghadam, P. Z.; Rogge, S. M. J.; Li, A.; Chow, C. M.; Wieme, J.; Moharrami, N.; Aragones-Anglada, M.; Conduit, G.; Gomez-Gualdron, D. A.; Van Speybroeck, V.; Fairen-Jimenez, D. Structure-Mechanical Stability Relations of Metal-Organic Frameworks via Machine Learning. *Matter* **2019**, *1* (1), 219–234. https://doi.org/10.1016/J.MATT.2019.03.002.

(18) Chen, C.; Ye, W.; Zuo, Y.; Zheng, C.; Ong, S. P. Graph Networks as a Universal Machine Learning Framework for Molecules and Crystals. *Chemistry of Materials* **2019**, *31* (9), 3564–3572. https://doi.org/10.1021/ACS.CHEMMATER.9B01294

(19) Ong, S. P.; Richards, W. D.; Jain, A.; Hautier, G.; Kocher, M.; Cholia, S.; Gunter, D.; Chevrier, V. L.; Persson, K. A.; Ceder, G. Python Materials Genomics (Pymatgen): A Robust, Open-Source Python Library for Materials Analysis. *Comput Mater Sci* **2013**, *68*, 314–319. https://doi.org/10.1016/J.COMMATSCI.2012.10.028.

(20) Willems, T. F.; Rycroft, C. H.; Kazi, M.; Meza, J. C.; Haranczyk, M. Algorithms and Tools for High-Throughput Geometry-Based Analysis of Crystalline Porous Materials. *Microporous and Mesoporous Materials* **2012**, *149* (1), 134–141. https://doi.org/10.1016/J.MICROMESO.2011.08.020.

(21) Hernández-Rivera, E.; Coleman, S. P.; Tschopp, M. A. Using Similarity Metrics to Quantify Differences in High-Throughput Data Sets: Application to x-Ray Diffraction Patterns. *ACS Comb Sci* **2017**, *19* (1), 25–36. https://doi.org/10.1021/ACSCOMBSCI.6B00142

(22) Jajko, G.; Calero, S.; Kozyra, P.; Makowski, W.; Sławek, A.; Gil, B.; Gutiérrez-Sevillano, J. J. Defect-Induced Tuning of Polarity-Dependent Adsorption in Hydrophobic–Hydrophilic UiO-66. *Communications Chemistry 2022 5:1* **2022**, *5* (1), 1–9. https://doi.org/10.1038/s42004-022-00742-z.

(23) Yu, Z.; Anstine, D.; Boulfelfel, S. E.; Gu, C.; Colina, C.; Sholl, D. Incorporating Flexibility Effects into Metal–Organic Framework Adsorption Simulations Using Different Models. *ACS Appl Mater Interfaces* **2021**, *13* (51), 61305–61315. https://doi.org/10.1021/acsami.1c20583.
