# Supplementary material for: Efficient Generation of Large Collections of Metal–Organic Framework Structures Containing Well-Defined Point Defects
Source: J Phys Chem Lett. 2023 Jul 18;14(29):6658–65. doi: 10.1021/acs.jpclett.3c01524 (PMC10388356; doi:10.1021/acs.jpclett.3c01524)
Supplement: Supplementary file 3 — jz3c01524_si_003.pdf [file jz3c01524_si_003.pdf]

jz-2023-01524x.R1

Name: Peer Review Information for "Efficient Generation of Large Collections of Metal-Organic Framework Structures Containing Well-Defined Point Defects"

First Round of Reviewer Comments

Reviewer: 1

Comments to the Author

The major advance reported in this paper is the tool that was created to readily and easily generate MOF crystals with missing linkers. The authors also provide the database of structures they generated. Through calculation of PXRDs and surface areas, the authors are also able to identify a threshold concentration of defects that would cause deviations in those quantities and would thus be easily detected in experiments.

There are multiple immediate significances to the advance. The first is the creation and dissemination of the tool to create new MOF databases that contain defects, allowing researchers to systematically interrogate defect effects on various tasks including but not limited to adsorption, as the authors have done. Another implication of this study is precisely the effect on adsorption that defects can have, especially when they're undetectable to experimental techniques. The concentrations of defects that can be achieved and still be undetectable can be appreciable for various structures. This suggests that much of the work in the literature that reports experimental results of adsorption could have been done, unknowingly, on defective structures. This can be part of the explanation behind the reproducibility issues of adsorption isotherms of MOFs that have been reported in the literature.

To aid in the discussion of how defects affect adsorption results, I suggest the authors add Henry's constants to the analysis as well as heats of adsorption to probe more how the interactions are affected by the presence of defects.

Lastly, a comment should be made on the fact that the missing linkers in the studied databases are not "defects", since they are not randomly distributed inside of the crystals. Instead, if I understand correctly, the missing linkers are repeated across the various images, so there is crystalline order. This means that they are creating a crystalline structure that has a missing linker, but that missing linker is repeated across the unit cells. A comment on this from the authors, given the focus of this study, is appropriate.

Author's Response to Peer Review Comments:

July 6, 2023

Prof. Editor
Senior Editor, Journal of Physical Chemistry Letters

Dear Prof. Editor,

I have submitted a revision of our manuscript jz-2023-0154x ("Efficient generation of large collections of metal-organic framework structures containing well-defined point defects") for consideration by *Journal of Physical Chemistry Letters*. The reviewer comments were positive and constructive. Below we have reproduced the reviewer comments and provided a point-by-point response. I hope you will find that this manuscript is now suitable for publication.

Thank you for your consideration,

David Sholl
School of Chemical & Biomolecular Engineering, Georgia Institute of Technology
Transformational Decarbonization Initiative, Oak Ridge National Laboratory

Response to Reviewers for jz-2023-0154x (Yu et al.)

We appreciate the positive and constructive comments from the reviewer of our manuscript. These comments are reproduced verbatim below, along with our point-by-point response.

Reviewer: 1
Recommendation: This paper is publishable subject to minor revisions noted. Further review is not needed.

The major advance reported in this paper is the tool that was created to readily and easily generate MOF crystals with missing linkers. The authors also provide the database of structures they generated. Through calculation of PXRDs and surface areas, the authors are also able to identify a threshold concentration of defects that would cause deviations in those quantities and would thus be easily detected in experiments.
There are multiple immediate significances to the advance. The first is the creation and dissemination of the tool to create new MOF databases that contain defects, allowing researchers to systematically interrogate defect effects on various tasks including but not limited to adsorption, as the authors have done. Another implication of this study is precisely the effect on adsorption that defects can have, especially when they're undetectable to experimental techniques. The concentrations of defects that can be achieved and still be undetectable can be appreciable for various structures. This suggests that much of the work in the literature that reports experimental results of adsorption could have been done, unknowingly, on defective structures. This can be part of the explanation behind the reproducibility issues of adsorption isotherms of MOFs that have been reported in the literature.
We appreciate the reviewer's positive assessment of our work.

To aid in the discussion of how defects affect adsorption results, I suggest the authors add Henry's constants to the analysis as well as heats of adsorption to probe more how the interactions are affected by the presence of defects.
We added the following text on p. 12 to explain why we did not include separate data on Henry's constants: "We report adsorption at a series of pressures for each molecule, and in each case the lowest pressure gives information about dilute loadings that can be considered in the Henry's limit.".

Lastly, a comment should be made on the fact that the missing linkers in the studied databases are not "defects", since they are not randomly distributed inside of the crystals. Instead, if I understand correctly, the missing linkers are repeated across the various images, so there is crystalline order. This means that they are creating a crystalline structure that has a missing linker, but that missing linker is repeated across the unit cells. A comment on this from the authors, given the focus of this study, is appropriate.
The reviewer is correct that the features introduced with our methods are in structures with periodic boundary conditions. We think it is still appropriate to refer to these features as defects, and this is consistent with much prior work on related materials, but we agree with the reviewer that it is important for readers to understand that these defects have long range order within the periodic crystal structure. We have added the following text on p. 6 to address this point: "Because the structures generated with our approach have periodic boundary conditions the

missing linker defects have long range crystalline order, even in cases where large computational volumes are used."

Additional Questions:
Urgency: High
Significance: High
Novelty: High
Scholarly Presentation: Top 10%
Is the paper likely to interest a substantial number of physical chemists, not just specialists working in the authors' area of research?: Yes

Formatting Requests from the Editorial Office

1) Title: In both the main manuscript file and the Supporting Information, set the title in title case, with the first letter of each principal word capitalized.

This update has been made.

2) Abstract: Shorten the abstract to 150 words or fewer.

The revised abstract has 148 words.

3) TOC Graphic: Please resize the TOC graphic per journal guidelines (2 in x 2 in).

The TOC Graphic was resized.

4) References: In both the main file and the supporting information, fix the style of all references to use JPCL formatting (check all references carefully). ***JPC Letters reference formatting requires that journal references should contain: () around numbers, author names, article title (titles entirely in title case or entirely in lower case), abbreviated journal title (italicized), year (bolded), volume (italicized), and pages (first-last). Book references should contain author names, book title (in the same pattern), publisher, city, and year. Websites must include date of access.

Response.

5) References: URLs are not preferred in reference citations becuase they lack permanence.

None of our references rely on URLs, although many include a DOI for convenience in addition to a complete citation.

6) Author Affiliations: Please include postal codes/country in the author affiliations in the publication file(s).

This information has been added.

7) Supporting Information: Please number SI pages in the following format: "S1, S2…"

The page number has been added.

8) Author List: Please use an asterisk to indicate the corresponding author(s) of your manuscript, and include their email address(es).

This information has been added.
